# Update on Retinoblastoma Therapies

**DOI:** 10.3390/medicina61071219

**Published:** 2025-07-04

**Authors:** Cristina A. Martínez Arce, Victor M. Villegas, Maura Di Nicola, Basil K. Williams, Timothy G. Murray

**Affiliations:** 1 School of Medicine, Universidad Central del Caribe, Bayamón 00960, Puerto Rico; 123cmartinez@uccaribe.edu; 2 Department of Ophthalmology, University of Puerto Rico, Medical Sciences Campus, San Juan 00936, Puerto Rico; 3 Bascom Palmer Eye Institute, University of Miami Miller School of Medicine, Miami, FL 33136, USA; mauradinicola@miami.edu (M.D.N.); basilkwilliams@med.miami.edu (B.K.W.J.); 4 Murray Ocular Oncology and Retina (MOOR), Miami, FL 33143, USA; tmurray@murraymd.com

**Keywords:** intra-arterial chemotherapy, intravitreal injections, intravenous chemotherapy, intracameral chemotherapy, periocular chemotherapy, enucleation, radiotherapy, prenatal screening, liquid biopsy, chemoplaque

## Abstract

Retinoblastoma is a success story in pediatric oncology, evolving from life-saving interventions to approaches that preserve eyes and vision while minimizing complications. Initially managed with enucleation and radiotherapy, treatment now emphasizes eye preservation through chemotherapy as the cornerstone therapy. Various chemotherapy delivery methods—including intravenous (IVC), intraarterial (IAC), intravitreal, intracameral, and periocular—offer flexibility in treatment. Studies show nearly 100% eye salvage rates for groups A–C. For advanced cases (groups D and E), IAC has achieved outcomes that were not possible before. Intravitreal injections, when performed safely, may help avoid enucleation and radiotherapy in advanced cases, preserving vision, even in complex scenarios, with vitreous seeding. Each strategy may be tailored to tumor and patient characteristics that may help optimize outcomes. Recent innovations like liquid biopsy, prenatal diagnosis, prognostic biomarkers, and new surgical methods, such as tylectomy and chemoplaque, are paving the way for more personalized care. While advanced extraocular or metastatic retinoblastoma remains challenging, these advancements underscore a shift towards better outcomes and individualized management. The future holds promise for refining treatment strategies to maximize eye and vision preservation while ensuring patient survival.

## 1. Introduction

### 1.1. Overview of Retinoblastoma

Retinoblastoma (RB) is the most common primary intraocular malignancy in children under five years old [1]. RB has an approximate incidence of 1 in 16,000 to 18,000 live births [2,3]. While there is no apparent predilection based on race, ethnicity, gender or laterality, significant disparities in RB outcomes persist across countries worldwide [4,5]. Lower-income regions experience markedly lower survival and globe salvage rates, highlighting the need for improved childhood cancer programs [6]. These disparities are due to many factors, in particular, a lack of early diagnosis and variety of treatment options [7]. Other considerations include late presentation with advanced disease, lack of awareness, treatment abandonment, required travel to a large treating center, and other limitations in providers, facilities, and equipment [8]. Cultural factors are also at play, such as the stigma associated with enucleation or the phenomenon of patients in certain countries opting for traditional treatment options, as opposed to seeking treatment from doctors [9,10]. In addition, the multidisciplinary efforts required for complex RB management can be significant. In many cases, the involvement of an ocular and pediatric oncologist, pediatric ophthalmologist, pediatrician, interventional radiologist or neurosurgeon, and ocular pathologist is key to a favorable prognosis [11].

RB arises due to mutations in the tumor suppressor RB1 gene on chromosome 13. As the model of Knudson’s “two-hit” hypothesis, RB can arise due to germline or somatic mutations. This hypothesis stated that two mutations (hits), one on each allele, are necessary for tumor development. In familial or congenital cases, the first “hit” arises in germline cells and the second “hit” is a somatic mutation. In comparison, both deletions in non-hereditary or sporadic RB are somatic mutations [12]. Familial RB is usually diagnosed earlier than sporadic RB [13]. Additionally, it is associated with a higher risk of developing secondary primary malignancies [14].

The complex genomic nature of RB increases the importance of genetic counseling. Patients with the RB1 gene mutation have a very high risk of transmitting the mutation to their offspring. Only 5% of patients with the mutation for RB do not develop the disease [15]. Therefore, all patients diagnosed with retinoblastoma should undergo genetic testing. If the patient is positive, both parents should be tested to determine if the mutation developed spontaneously in the patient during embryogenesis or if it was inherited from a parent. If genetic testing is not performed, “at-risk” patients, defined as a parent, sibling, or a first- or second-degree relative of a patient affected by RB, should undergo screening via ophthalmic examination. Depending on genetic testing and the relationship to the affected family member, a risk category is assigned to the “at-risk” patient. Babies at high risk are recommended to receive early and frequent screening through a dilated fundus examination that is eventually spaced out in time until they are 7 years old. If they have a known RB1 mutation, screening should be continued every 1 to 2 years [16]. It is important to note that genetic testing for retinoblastoma (RB) is not 100% sensitive, and a very small percentage of patients with a negative test result may still have the disease. Therefore, it is crucial to interpret negative results with caution.

**Figure 1:**
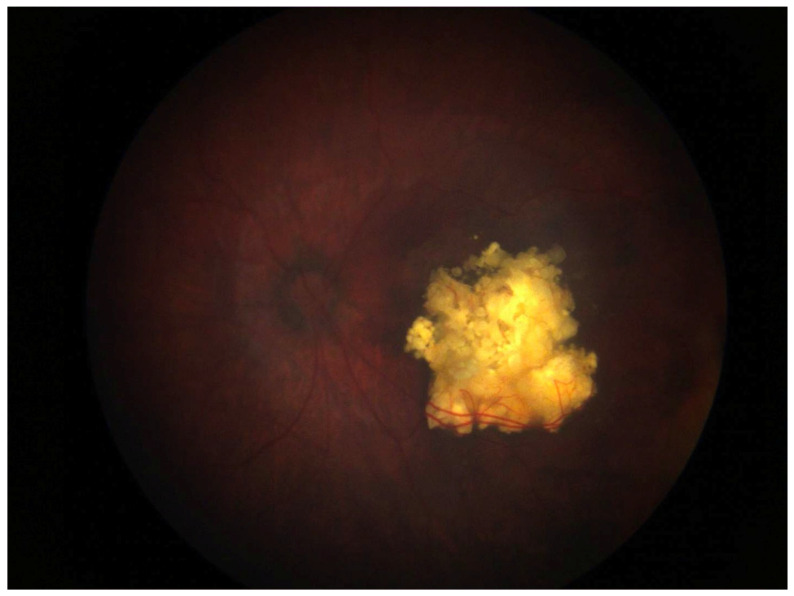
Regressed endophytic retinoblastoma after intra-arterial chemotherapy.

RB is characterized by a wide range of signs and symptoms. RB should be heavily considered as the possible diagnosis for any white or yellow lesion in the posterior segment of children under the age of 5. It is typically identified by a creamy white retinal mass, which may be unilateral or bilateral and identified by leukocoria. Other signs may include strabismus, nystagmus, and red eye. RB tumors tend to be painless, unless there is associated aseptic orbital cellulitis or neovascular glaucoma with hemorrhage [17,18]. Tumors can demonstrate endophytic growth, toward the vitreous, exophytic growth, toward the choroid, or a combination of both. Endophytic growth may result in vitreous seeding while exophytic growth can cause an exudative detachment and subretinal seeding [19] (Figure 1).

### 1.2. RB Staging and Classification

RB has been categorized in different ways for the purpose of identifying appropriate treatment strategies. Classification focuses on the eye (intraocular disease), while staging takes into account the whole body (extraocular extension).

The Reese and Ellsworth (R–E) system, published in 1964, classified intraocular RB and stratified eyes according to their risk of enucleation with external beam radiation. After intravenous chemotherapy was introduced in the 1990s, a new classification system, the International Intraocular Retinoblastoma Classification (IIRC), was developed to describe disease in groups A to E, according to its size, location, and other characteristics. Group A tumors have the highest likelihood of globe salvage due to their small size, location limited to the retina, and a lack of vitreous or subretinal seeding. In contrast, Group E eyes usually have been significantly compromised anatomically and rendered dysfunctional (Table 1) [20]. A similar classification system was introduced in 2005, the Intraocular Classification of Retinoblastoma (ICRB), which modified the specifications for groups D and E [21].

Extraocular RB is typically seen in low- and middle-income countries. It involves a tumor that extends outside the globe, is visible around the eye, and has a high risk of metastasis [20]. For extraocular RB, Chantada et al. [22] designed the International Retinoblastoma Staging System (IRSS), which categorizes the disease from stages 0 to IV. Stage 0 indicates a patient whose disease is still intraocular and is treated conservatively, with non-invasive, less-aggressive methods, which result in a positive prognosis. In contrast, Stage IV describes a patient whose RB has metastasized, either via the central nervous system or the bloodstream. Another way to categorize extraocular RB is through the American Joint Committee on Cancer (AJCC) Staging System. With the aim of predicting survival rather than eye salvage, the tumor is staged is based on its intraocular extent (T), regional lymph node involvement (N), and distant metastasis (M). This system factors in genetic status as well by including an H modifier which indicates heritable (H1) or non-heritable (H0) disease [23].

### 1.3. Purpose of the Review

The purpose of this review is to summarize the current therapeutic approaches (strategies) for RB and evaluate their outcomes and efficacy. We will also highlight the role of recent advancements which provide alternatives for less invasive treatment methods. Finally, we will discuss key findings from pioneering research that are shaping the future of RB management.

## 2. Methods

We conducted a targeted literature search using PubMed and Google Scholar, supplemented by manual screening of reference lists from relevant articles. We used the following keywords: “intra-arterial chemotherapy”, “intravitreal injections”, “intravenous chemotherapy”, “intracameral chemotherapy”, “periocular chemotherapy”, “enucleation”, “radiotherapy”, “prenatal screening”, “liquid biopsy”, and “chemoplaque”. To comprehensively identify relevant literature, we included additional terms that addressed the etiology of RB and management strategies. The selection prioritized primary research articles published between 2015 and 2025, with relevant review articles to provide context for emerging concepts.

## 3. Therapeutic Approaches

To successfully treat RB, a multidisciplinary approach is needed. The main focus of management should be saving the patient’s life, followed by aiming for eye preservation and maximum visual function. Among the many available treatment modalities, systemic chemotherapy currently plays a central role, especially for intraocular RB. The most commonly approved chemotherapy protocol is CEV therapy, a combination of carboplatin, etoposide, and vincristine, administered in 6 cycles throughout 6 months [24]. With this regimen, a high efficacy in tumor control can be achieved across various disease groups. Systemic chemotherapy is often followed by local consolidation therapies, such as plaque radiotherapy or cryotherapy, to enhance therapeutic outcomes. Additionally, intra-arterial chemotherapy, most commonly applying melphalan, has emerged as an effective targeted treatment alternative, as it delivers drug concentrations directly to the eye with reduced systemic toxicity [25]. These protocols form the backbone of current RB management and are supported by extensive clinical evidence and international guidelines.

### 3.1. Early Treatments

#### 3.1.1. Enucleation

The first treatment for RB was enucleation, and this remained the predominant approach throughout the 1800s and early 1900s. Even though many countries have shifted towards globe-saving procedures, the technique for enucleation has improved to reduce associated morbidity and increase survival rates. Enucleation remains a life-saving procedure, with reported 5-year survival rates exceeding 90% in cases where the tumor is confined intraocularly and treated promptly [26]. However, survival decreases significantly when there is extraocular extension or metastatic disease [27]. In most cases, it is reserved for advanced RB when there is little to no chance of eye preservation, typically with regard to group D and E tumors with extraocular extension, poor tumor visualization, buphthalmos, pthisis bulbi, neovascular glaucoma, anterior chamber seeding, and diffuse infiltrating RB [28,29]. Primary enucleation is typically used in the cases of severe unilateral intraocular RB. Secondary enucleation is used for persistent or recurrent tumors when conservative treatment has failed or in nonfunctional or painful eyes after high-dose chemotherapy [30].

While enucleation effectively controls the primary tumor, it is associated with potential complications including socket contracture, implant exposure or migration, and cosmetic deformities, which may affect quality of life [31]. Additionally, the psychological impact due to loss of eye can be significant, especially in children. Enucleation may be a treatment option for other non-malignant conditions, such as severe ocular trauma, intraocular infection, and phthisis bulbi, but applying this procedure in patients with RB requires specific surgical precautions. It is essential to obtain the largest possible nerve stump to ensure accurate staging and reduce the risk of extraocular spread. The removal of a minimum of 15 mm of optic nerve stump is recommended in all cases [32]. Advances in surgical technique and postoperative care have helped minimize morbidity, but long-term surveillance remains critical to detect any metastatic progression.

#### 3.1.2. Radiotherapy

Another early treatment for RB management was radiation therapy, which uses high energy radiation to destroy tumor cells in the affected eyes. Different types of radiotherapy have been implemented and refined throughout the years. External beam radiotherapy (EBRT) was the first used to control RB tumors. However, treatment has shifted away from EBRT because of its severe side effects, including mid-face hypoplasia, cataracts, radiation retinopathy, optic nerve damage, and an increased risk of second primary malignancies in the field of radiation. Currently, EBRT is reserved for high-risk tumors that do not respond to other treatments but where enucleation is not appropriate, like a monocular patient with active disease in the only remaining eye [33].

Although EBRT is no longer a first-line treatment, due to the risk of severe ocular complications and a 36–51% risk of second cancers in heritable retinoblastoma, it continues to serve as a salvage option after failure of chemotherapy and focal therapy. In one study, the overall ocular salvage rate was 46%, with control rates ranging from 25% to 100% depending on tumor characteristics. Vitreous seeds at the time of EBRT and tumor stage migration during chemotherapy were significant predictors of poor response, whereas factors like gender, tumor site, laterality, number of chemotherapy cycles, and initial RE stage were not predictive. Notably, tumor control was still achieved in select cases, including those with prior treatment failure, reinforcing the role of EBRT as a targeted salvage strategy when carefully indicated [34].

Novel teletherapy techniques serve as alternatives for treating high-risk large RB tumors. For example, intensity modulated radiotherapy (IMRT) and stereotactic radiotherapy aim to use a hypofractionated dose schedule to achieve a conformal dose distribution to the eye tumor. Compared to EBRT, reports state that IMRT has an improved dose conformity and a reduction in radiation exposure to surrounding normal tissues, which may potentially lower the risk of some adverse effects. However, understanding of long-term survival and toxicity data specific to IMRT remains limited, spiking concerns about radiation-induced secondary malignancies and warranting further research [35]. Additionally, advanced RB can be treated with proton beam therapy (PBT), which aims to decrease the radiation dose to the bone surrounding the eye to reduce the risk of secondary cancers. PBT achieves local control rates comparable to EBRT while significantly reducing the risk of radiation-induced secondary malignancies. In long-term follow-up studies with median durations around 8 to 13 years, no radiation-associated malignancies were observed, although more data is still necessary to fully confirm this advantage [36]. Therefore, this procedure has shifted to a salvage treatment rather than a first-line method [37].

Plaque radiotherapy is a type of focal therapy that does not present an increased risk of second primary malignancies. It has proved useful in the treatment of group A, B, and C RB, provided the tumors are small, isolated, and far from the optic nerve or macula, or for tumor recurrence [33]. Shields et al. [38] found that this procedure was especially effective for tumors that failed prior treatments like chemotherapy or laser therapy. Age and the presence of seeding increased the likelihood of tumor recurrence, while local complications occurred, but no secondary malignancies were reported.

### 3.2. Shift in Treatment Approaches

The implementation of enucleation and radiotherapy has gradually decreased throughout the years, as chemotherapeutic approaches not only focus on saving the patient’s life but also preserving vision with a better side effects profile [39].

### 3.3. Chemotherapy

#### 3.3.1. Intravenous Chemotherapy

Intravenous Chemotherapy (IVC), also called chemoreduction, was developed in the 1990s. Recently, in high income countries, the use of IVC has been selectively preferred in patients with bilateral disease, confirmed germline mutation, family history of RB, or cases with suspected optic nerve or choroidal invasion [40]. This procedure applies the use of two to four chemotherapeutic agents, usually vincristine, carboplatin, and etoposide, administered through a peripheral or central catheter. Treatment is performed monthly and consists of three to nine consecutive cycles [41]. Notably, recent studies have shown that in patients with unilateral pathologic high-risk RB, three cycles of CEV (carboplatin, etoposide, and vincristine) chemotherapy provide 5-year disease-free survival rates comparable to the traditional six-cycle regimen. This suggests that shorter treatment courses may be equally effective while potentially decreasing toxicity and treatment burden [42].

The long-term effects of IVC have been evaluated and support its efficacy in tumor control of less severe cases (groups A to C). More advanced cases require the addition of other therapies, such as IAC or plaque radiotherapy, which have been shown to improve the ESR by 5% to 28%. Pinealoblastoma, metastasis, and death are rare complications of this procedure [43]. Additionally, IVC may also be co-administered with intra-arterial chemotherapy (IAC). This procedure is known as ASIAC (Alternate Systemic IVC and IAC) and, although used sparingly, it may be preferred when trying to increase eye salvage rate (ESR). Han et al. [44] applied this technique and achieved a 100% ESR for low-risk RB and >60% salvage rate for high-risk RB, compared to the estimated 40–60% ESR for IAC alone. Changes in treatment, such as including a two-drug regimen of IAC and intravitreal chemotherapy (IviC), may have increased the ESR. However, further research is needed to identify the possible complications of this procedure.

#### 3.3.2. Intra-Arterial Chemotherapy

In the 2000s, IAC was introduced as a treatment for intraocular RB. This procedure focused on the catheterization of the ophthalmic artery to deliver chemotherapeutic drugs, such as melphalan, topotecan, and carboplatin, into the eye. This method results in decreased systemic toxicity and direct delivery of a high chemotherapeutic dose to the tumor [25]. Due to its success, it is now the preferred treatment in high-income countries for advanced RB (group D and E), specifically unilateral RB [45,46]. However, poor visualization of the tumor, extraocular extension, or abnormal cerebral vasculature may contraindicate the use of IAC [47].

Even though the systemic side effects are reduced compared to other RB treatments, some are still of concern. For example, Lee et al. [48] studied the presence of neutropenia after IAC treatment and found an incidence of 40% in their study sample. The authors suggested that topotecan is associated with neutropenia and limiting its dose, with an accompanying high dose of melphalan, may reduce the risk of this systemic effect. Bach et al. [49] reported three cases of adverse effects from melphalan. All cases were group D unilateral RB without metastasis. The side effects reported were: 1. total hearing loss in the ipsilateral ear; 2. cutaneous periauricular erythema; and 3. extravasation of the chemotherapeutic agent resulting in proptosis and infiltration, and thickening of the medial rectus.

Other possible complications of IAC include ophthalmic artery vasospasm, retinal or choroidal ischemia, and retinal detachment [50]. Rarely, IAC may lead rhegmatogenous retinal detachment due to rapid tumor progression, especially in patients with Group E RB, endophytic tumor configuration, and extensive vitreous seeding [51]. Exudative retinal detachment could also result from IAC and may require removal if it persists with vision loss and loss of tumor control [52]. Groin hematoma and ipsilateral eyebrow or eyelash loss have also been reported as side effects of IAC [53]. Self-limited skin erythema and swelling of the periocular have also been observed in patients who have received IAC [54].

#### 3.3.3. Intravitreal Chemotherapy

IViC was initially proposed as a treatment method in the 1960s but was not widely accepted until the 2010s due to concerns regarding extraocular extension of the disease. This technique aims to treat advanced intraocular RB, specifically when vitreous seeds are present, as their avascular nature makes them more difficult to treat with other forms of chemotherapy that are brought to the eye through the vascular system. The difficulty of achieving tumoricidal concentrations of the chemotherapeutic agents in the affected eye compartments accounts for the lack of response of RB seeding to many types of treatment. However, injections of chemotherapeutic agents directly into the vitreous cavity have been shown to achieve the adequate drug concentration targeted at the tumor, with some risk of retinal toxicity [55].

Initially, melphalan was used as a first-line treatment with IViC. Although it was shown to provide successful control of vitreous seeding in some patients, high doses of this agent were contraindicated due to its toxicity [56]. Cieślik et al. [57] assessed the concentration of melphalan currently used for IViC and the recurrence of RB with seeding. The authors found that reducing the dose of melphalan to decrease the risk for retinal toxicity resulted in a higher recurrence rate of RB in the following months. The current recommend dose is 30, with an initial dose of 40 μg for more advanced cases. Doses of 20 μg are deemed insufficient.

Topotecan was then combined with melphalan for the successful treatment of recurrent vitreous seeds, which resulted in a favorable safety profile with mild complications from this use of this new agent, such as temporary hypotonia, epithelial defect, and vitreous hemorrhage [58]. Furthermore, the use of topotecan as a standalone medication has been studied due to its reduced side effect profile and potential benefit for retinal tumors and subretinal seeding. Bogan et al. [59] compared the efficacy of topotecan to melphalan in rabbits and found that topotecan caused no retinal toxicity, while melphalan caused reduced electroretinography (ERG) amplitudes of up to 79%, resulting in dose-dependent retinal toxicity. The authors found no significant ERG reductions in patients treated with topotecan and a 100% eye salvage rate in those treated exclusively with this chemotherapeutic agent.

#### 3.3.4. Periocular Chemotherapy

Periocular chemotherapy consists of an injection of a chemotherapeutic agent, usually topotecan or carboplatin, into the tissues around the eye. The agent is most commonly injected into the sub-tenon’s space, but depending on the technique and clinical goal, the subconjunctival space may be preferred [60]. The chemotherapeutic agent is then absorbed through the sclera and cornea. Currently, topotecan is the preferred agent due to its lower toxicity [61]. Sthapit et al. [62] demonstrated that periocular topotecan can be administered alone or in combination with the triple agent IVC for the effective management of vitreous seeds in RB. They detailed that 63% of the eyes that underwent the therapy had complete regression of vitreous seeds. More persistent vitreous seeds needed further treatment with other techniques.

Sub-tenon’s carboplatin was initially introduced in the 1990s, but was later abandoned because of reports of orbital fibrosis and subsequent restriction of eye movement [63]. Although it was a potentially less invasive option for the treatment of vitreous seeding, the use of safer and more effective methods, such as IViC, outweighed the risks of this procedure. This method showed potential because a combination of local and systemic chemotherapy may optimize drug exposure and reduce side effects. Nemeth et al. [64] found that combining subconjunctival carboplatin and systemic topotecan was more effective than systemic carboplatin and subconjunctival topotecan in rats with orthotopic xenografts. In a subsequent longitudinal study, the preferred treatment eliminated RB in mice and restored vision in some survivors. However, this may not be applied in clinical practice due to available treatment alternatives.

#### 3.3.5. Intracameral Chemotherapy

As a recent innovation, intracameral chemotherapy (ICC) has been used for treating RB with aqueous seeding (AS), which is common in two subtypes of RB: diffuse infiltrating and diffuse anterior RB [65,66]. Primary AS is part of group E RB [21], while secondary AS is typically reported during conservative management and is a sign of treatment failure. ICC treats aqueous invasion through a series of bicameral injections of the chemotherapeutic agent into both the anterior and posterior chamber of the eye. Where intravenous, intravitreal, and periocular therapies have failed to reach the adequate drug concentration in the aqueous, ICC may cause sufficient regression of AS, improve long-term survival, reduce toxicity, and prevent metastasis [67].

In 2016, Munier et al. [68] reported the first successful use of intracameral chemotherapy for AS in RB. Stathopoulos et al. [69] subsequently presented a larger series of primary or secondary non-iatrogenic AS treated with ICC with melphalan or topotecan, demonstrating complete response of AS after 1 month with an average four or five injections, depending on the agent. There was AS recurrence in only three cases, which all resolved with additional treatments of ICC. Additionally, 22% of the grouped cases were classified as group B RB, suggesting that AS is not limited to advanced RB diagnoses or that there may be limitations in the current classification system. Though not widely adopted as a standard procedure of ICC, Borroni et al. [70] presented an alternate one-step approach to aspirate the aqueous and apply melphalan in a single injection to treat group E RB with anterior chamber seeding. A system of a three-way cannula and cryotherapy were applied simultaneously as the agent was injected, with the purpose of reducing the risk of tumor cell dissemination.

### 3.4. Surgical Methods

#### 3.4.1. Tylectomy

An alternative treatment that has been performed is tylectomy, or the surgical resection of intraocular RB. Though its use remains disputed, Zhao et al. [71] documented that patients that underwent tylectomy had a higher 5-year disease-specific survival than patients who underwent other eye salvage procedures. Thus, the authors suggest that it is a good alternative for chemotherapy-resistant RB, since it eliminates the tumor regardless of its biology. Likewise, vitreous opacity and neutropenia induced by chemotherapy, which may affect eye salvage outcomes, may be prevented if tylectomy is performed instead. However, tylectomy remains controversial and is not recommended as a primary therapy.

#### 3.4.2. Chemoplaque

A chemoplaque consists of a reservoir capable of containing chemotherapy, which is glued to the scleral wall to allow for a slow and continuous release of chemotherapy into the eye. From 2020 to 2024, Gallie et al. [72] conducted clinical trials to test the safety and efficacy of the novel sustained-release topotecan episcleral delivery system (chemoplaque) in patients with intraocular RB in at least one eye after first-line therapy. Although more time is needed to understand the efficacy and side effect profile, the chemoplaque technique shows promise for future treatment strategies.

The following table (Table 2) provides a summary of the treatment options available for RB and lists the most common adverse effects associated with each. This outline may provide a concise reference to support clinicians in selecting appropriate RB therapy.

## 4. Diagnostic and Prognostic Advancements

### 4.1. Prenatal Diagnosis

Families with a germline RB1 mutation may undergo genetic counseling and opt for prenatal diagnosis of RB. A pre-implantation technique via in vitro fertilization may be considered during family planning. This method aims to detect a mutated RB1 gene before transferring an embryo and provides an accurate diagnosis, allowing couples to choose an embryo without the mutation. Post-implantation, non-invasive early prenatal screening may be considered. These are done at 8 weeks of gestation by evaluating the cell free fetal DNA material in the mother’s blood. Alternatively, an invasive early prenatal test may be performed, including chorionic villi sampling at 11 weeks or amniocentesis at 16 weeks. Alternatively, late prenatal screening, done during the third trimester, includes fetal ultrasounds or MRIs, which may be diagnostic if tumors are already present in utero but does not indicate whether RB will develop in the future [73].

Lewis et al. [74] evaluated the vasculature of an RB tumor in utero, applying the use of microflow imaging for prenatal screening of RB. Fetal screening with ultrasound identified a tumor at 32 weeks’ gestation and the tumor bed vasculature was visible on microvascular flow imaging. Since frequent monitoring revealed tumor growth, the child was induced at 37 weeks and treated with systemic carboplatin. The authors concluded that microvascular flow imaging is an effective, non-invasive method.

### 4.2. Prognostic Biomarkers

Prognostic biomarkers in RB are used to predict the progression of the disease, response to treatment, and survival outcomes. Histopathological markers, such as choroidal, optic nerve or scleral invasion, indicate an increased risk of metastasis or poor prognosis [75]. Genetic markers like RB1 mutations are used to determine hereditary risk and bilateral disease, while MYCN amplification is characteristic of aggressive, sporadic, unilateral cases [76]. Clinical markers include IRCB classification and AJCC staging systems.

Seigel et al. [77] studied RB cells lines and found overexpression of RNA nuclear transporter protein ALYREF/THOC4 within the tumor, optic nerve, and adjacent retina, similar to the overexpression of certain proteins seen in other cancers. Due to its pattern of expression, the authors suggest that this protein may have a role in optic nerve invasion and metastasis of RB. However, this is not a standard prognostic marker, since further studies are required to clarify the role of ALYREF in RB pathology. Other newly studied biomarkers include miRNAs. MiR-20a, miR-135a, and miR-191 specifically have been recognized as potential markers to distinguish invasive from non-invasive RB. These miRNAs were elevated in invasive cases, which suggest a link to tumor aggressiveness and their use as potential biomarkers [78].

### 4.3. Liquid Biopsies

A liquid biopsy is a non-invasive method used to monitor RB through the analysis of tumor-derived genetic material. It consists of detecting tumor markers, such as circulating tumor DNA (ctDNA), cell-free DNA (cfDNA), circulating tumor cells (CTC), tumor educated platelets (TEP), extracellular vesicles, proteins, and metabolites in body fluids. Plasma (serum) and aqueous humor (AH) are commonly used for the diagnosis, prognosis, and genetic analysis of RB. This method is particularly important for RB due to the risk of extraocular tumor spread associated with direct tumor biopsy [79]. This procedure is, overall, well-tolerated and has very few risks [80]. A test for confirmation of diagnosis via liquid biopsy is now clinically available at Children’s Hospital Los Angeles (CHLA).

The AH biopsy represents a significant milestone in ocular oncology, providing tumoral genomic data that can be collected at diagnosis and repeatedly throughout treatment. This enables dynamic insights into disease progression and treatment response. Liquid biopsy of the AH contains detectable levels cfDNA. Comparing the cfDNA content before and after enucleation reveals strong genetic correlations. Serial monitoring of somatic copy number alterations (SCNAs) in AH demonstrates reductions during tumor regression and increases during recurrence, indicating a potential in disease monitoring. A gain of chromosome 6p was found more frequently in enucleated eyes compared to salvaged eyes. This, along with MYCN amplification, was linked to a worse prognosis. Additionally, cfDNA in AH reflects the overall intraocular genetic environment, which can differ significantly between eyes, even in bilateral disease [81]. The potential to use liquid biopsy as a first-line diagnostic test and for early detection of recurrence or incomplete response marks it as a pivotal advancement in the RB management.

Abramson et al. [82] suggested that detecting alterations in circulating tumor RB1 DNA (ctRB1) may be useful for monitoring treatment response. The authors demonstrated that ctRB1 levels progressively decreased within minutes of severing the optic nerve during enucleation. Thus, these findings suggest that the half-life of ctRB1 shortened after the procedure. This is clinically significant because, in other cancers, a decrease in ctDNA after treatment has been associated with a better prognosis [83].

## 5. Outcomes and Efficacy

### Eye Salvage Rates

An eye salvage rate (ESR) is the ratio of eyes salvaged to the total number of eyes treated. The value is typically reported based on the treatment approach and RB group treated in each case. Although a standard ESR value for RB prognosis has not yet been established, Daniels et al. [84] carried out a meta-analysis that summarizes key insights into the ESRs associated with IVC. The authors reviewed studies that used Reese–Ellsworth or IIRC classification systems, and, regardless of the system, globe salvage rates consistently decreased with increasing disease severity. Older patient age was also identified as a predictor of poorer prognosis for eye salvage, regardless of treatment type. Additionally, the authors considered the outcomes of different treatment regimens regarding eye salvage. Compared to standard regimes of IVC, two-drug regimens resulted in lower globe salvage rates across all disease severities. In contrast, approaches that add a fourth drug, extra cycles, or second-line agents increased eye salvage rates in advanced disease. Thus, the authors suggest that a higher-intensity regimen may be considered in these cases, if IAC cannot be performed. However, they did not discuss the possible side effects, which must be considered if this is applied clinically.

Other treatment methods, such as IAC, have been associated with different ESRs. Although the values vary throughout studies, there has been consensus that IAC leads to higher ESRs than other methods [85]. However, since IAC coincided largely with the introduction of IViC, it is challenging to assess whether the improved outcomes are due to IAC alone or a combination of IAC and IViC. Although IViC has historically been considered insufficient as a standalone treatment for RB, many studies have evaluated globe salvage rates after therapy with IAC, IViC, and a combination of both.

Dalvin et al. [86] reported a 100% eye salvage rate after IAC treatment (mainly with melphalan) in patients with group B and C RB. For groups D and E, they did not report a significant difference in eye salvage rates between groups treated with IAC alone or a combination of IAC and IViC (with melphalan and topotecan), with eye salvage rates ranging from 57% to 88%. Mirzayev et al. [87] evaluated patients from groups B to E and reported eye salvage rates of 50% for patients that underwent secondary IAC. They also reported a 70% eye salvage rate in their IViC group and 71.4% in their combined group. For IAC they used, in all patients, melphalan and added topotecan for more severe cases. For IViC they initially used melphalan alone and in later phases of the study adopted a combination of melphalan and topotecan.

## 6. Challenges and Limitations

### 6.1. Advanced Extraocular or Metastatic Retinoblastoma

Extraocular RB occurs when there is extension of tumor to soft tissues surrounding the eye or to the optic nerve. Once RB extends even further into the body, it is termed metastatic RB. The most common site of metastasis is the central nervous system (CNS), and the most common route is through the optic nerve [88]. Metastatic RB poses new challenges for treatment because once the disease has spread, the prognosis quickly worsens, especially when the CNS is involved. Advanced clinical stage, large tumor size, and high-risk ocular features, such as hemorrhage, cellulitis, and glaucoma, are also associated with poor prognosis and low survival rates of metastatic RB [89]. First-line treatment is currently systemic multi-agent chemotherapy. However, this treatment results in severe adverse effects, such as myelosuppression, risk for infections, and need for blood transfusions [90]. High-dose chemotherapy with stem cell rescue has also been presented as an alternative, but survival is still low [91].

The rising role of immunotherapy is transforming the treatment perspective for metastatic retinoblastoma, offering promising new strategies that leverage the immune system to target-resistant disease. New approaches for metastatic RB treatment include using chimeric antigen receptor (CAR) T-cells to target glypican-2 (GPC2), which is specifically expressed in RB cells and not in mature, healthy tissues. Tested on mice, this method significantly regressed the brain tumor, delayed spinal cord spread, and prolonged mouse survival. Although further evaluation to assess side effects is required, GPC2 CAR T-cells could serve as a foundation for future clinical treatments of metastatic RB [92].

Another treatment strategy for extraocular RB with CNS involvement was anti-GD2 monoclonal antibody (mAb) naxitamab following reduced intensity myeloablative chemotherapy and autologous stem cell transplant (ASCT), along with intrathecal topotecan [93]. The authors reported success in two cases of bilateral, metastatic RB with minimal disseminated disease detected in the cerebrospinal fluid. These patients showed no signs of RB for over 4 years, which highlights the potential of naxitamab to contribute to long-term survival. Importantly, intrathecal topotecan was effective in clearing CRX-positive minimal disseminated disease in the CSF, underscoring the value of integrating molecular diagnostics with targeted CNS-directed therapies to improve outcomes in high-risk metastatic RB.

Eichholz et al. [94] provided clinical data that demonstrated that anti-GD2 mAb dinutuximab beta shows promising activity in patients with relapsed stage IV RB following autologous stem cell transplantation. Some patients achieved sustained remission beyond two years, but relapses were reported, emphasizing the need for constant follow-up and the possibility of combining therapies to enhance maintenance. Complementary in vitro studies demonstrated that anti-GD2 and anti-B7-H3 antibodies can effectively kill retinoblastoma cells.

### 6.2. Other Clinical Considerations

Refractive errors, including hyperopia and astigmatism, are more prevalent in patients with RB, contributing to an increased risk of amblyogenic factors [95,96]. Additionally, RB patients are more likely to present with strabismus secondary to decreased vision, which presents another significant amblyogenic risk factor [97]. Given that approximately 40% of RB cases are hereditary, due to RB1 germline mutations, early identification of visual defects is critical [98]. Regular ocular screening and timely intervention are essential not only for preserving vision but also for monitoring for associated complications such as pineoblastoma.

## 7. Future Directions

### 7.1. Innovations in Treatment

Photodynamic therapy (PDT) consists of administering photosensitizing molecules that are activated when they are exposed to light of different wavelengths, thus triggering their antitumor effects. This technique could be a treatment option for retinoblastoma, because it consists of a targeted, non-mutagenic mechanism, which only activates antitumor effects when exposed to the light of an adequate wavelength. Thus, the damage to surrounding healthy tissue is minimal [99]. Even though several studies have been conducted to assess this procedure, it has not been applied widely as a standard treatment approach for RB. However, this method provides the possibility of targeting tumor cells, while minimizing damage to surrounding healthy tissues [100]. Recent studies focus on various photosensitizers and light delivery methods. Zhou et al. [101] demonstrated the efficacy of TPE-IQ-2O, a mitochondria-targeting photosensitizer, in mice. They concluded that TPE-IQ-2O demonstrates strong antitumor activity with its notable capacity for generating reactive oxygen species and lack of significant toxicity. The authors propose that this mitochondria-specific PDT strategy could significantly decrease the risk of metastasis. However, further research is required to fully understand if PDT can be successfully applied to humans with RB.

Gene therapy is the process of introducing a healthy copy of a gene to correct defective genes in an individual. The success of gene therapy depends largely on the use of effective vectors [102]. This approach directly targets the underlying RB1 gene mutation, potentially providing more precise, less toxic, and longer-lasting treatment compared to conventional therapies. Although it has been studied to a limited extent regarding RB, recent advancements suggest it holds promise for future treatment. In 2019, Pascual-Pasto et al. [103] studied the possible application of VCN-01, an adenovirus vector, in RB gene therapy. The authors tested the intravitreous administration of VNC-01 in mice and rabbits with RB and found favorable results. There was improved eye survival and prevention of metastasis to the brain, along with negligible side effects. Mandal et al. [102] reviewed the proposed use of nanoparticles, which are biocompatible, but they may interact with surrounding tissues and cause toxicity. In the search of effective vectors, Haase et al. [104] coupled atrial natriuretic peptide (ANP) with hyaluronic acid-coated gold nanoparticles (HA-GNPs) with the purpose of combining the HA coat’s high ocular delivery rate with ANP’s antitumorigenic features. The authors effectively demonstrated the reduction of RB tumor growth in mice models. While gene therapy shows considerable potential in preclinical models, its role in the treatment of RB remains to be clarified through future studies in human patients.

### 7.2. Management and Surveillance

Many recent advances have paved the way for earlier diagnosis of RB and more effective treatment, as each discovery drives the development of more targeted and less toxic therapies. However, there is no set standard of care for applying these therapies. Personalized treatment approaches play an important role, requiring case-by-case evaluation to avoid unnecessary interventions. Eye salvage rates and patient survival are difficult to predict due to lack of resources, and variable and complex presentations of the disease.

Monitoring survivors of RB requires long-term follow-up strategies, which are at the discretion of the attending physician and vary widely throughout care centers. Surveillance recommendations, such as annual follow-up, are advised for early detection of possible complications. For example, due to high risk of melanoma, routine dermatologic check-ups are recommended. In addition, sinusitis, pain, and skeletal tenderness are symptoms that should not be overlooked and require further evaluation. Rb survivors are also at high risk for bone and soft tissue sarcomas, but the use and effectiveness of whole-body MRI is still debatable [105].

## 8. Conclusions

Breakthroughs in RB management have been promising and focused on the main goal of patient survival. Novel treatment strategies aim to improve globe salvage rates and maximize vision, while maintaining high rates of survival. Intravitreal, periocular, and intracameral chemotherapy address advanced stages of RB, including seeding and aqueous invasion, yet their true efficacy remains uncertain. Liquid biopsies are also favorable as a non-invasive method for early detection and monitoring of RB. Gene therapy and photodynamic therapy are likely to be refined with future advancements and may lead to improved outcomes. However, there are still many challenges, especially when treating cases of metastatic and extraocular RB, where the prognosis remains poor. Thus, continued development of more targeted treatments is still needed. Ongoing research and clinical trials are key for refining treatment regimens and improving survival rates. A multidisciplinary approach, incorporating innovative therapies, advanced diagnostics, and personalized treatment, remains essential in advancing the management of retinoblastoma.

## Figures and Tables

**Table 1:** International Intraocular Retinoblastoma Classification.

Groups	Description	Clinical Features
A	Very low risk	Small tumors (<3 mm), confined to the retina, ≥3 mm from foveola and ≥1.5 mm from optic disc. No subretinal fluid or seeding.
B	Low risk	Larger tumors (>3 mm), any location not in Group A. May have subretinal fluid ≤ 5 mm from tumor base. No subretinal or vitreous seeding.
C	Moderate risk	Localized seeding: minimal vitreous/subretinal seeds close to the tumor. Discrete tumor of any size and location. Subretinal fluid confined to one quadrant may be present.
D	High risk	Diffuse seeding (massive vitreous or subretinal seeds) and/or massive, non-discrete endophytic or exophytic disease. More widespread seeding than Group C. May present with greasy-appearing vitreous seeding or avascular masses. Subretinal seeding may be plaque-like.
E	Very high risk	Extensive tumor that has destroyed the eye anatomically or functionally with at least one of the following: Intraocular neovascular glaucoma Massive intraocular hemorrhage Aseptic orbital cellulitis Tumor anterior to anterior vitreous face Tumor touching the lens Diffuse infiltrating RB Phthisis or pre-phthisis

**Table 2:** Summary of treatment methods for RB and side effects.

Treatment	Procedure	Adverse Effects
Enucleation	Surgical removal of the affected eye when other treatments fail.	Loss of vision Surgical risks Psychological impact Fascial asymmetry
Radiotherapy	Use of external beam or plaque radiation to destroy tumor cells.	Cataracts Dry eye Orbital hypoplasia Secondary cancers Radiation retinopathy and neuropathy
Intravenous Chemotherapy (Chemoreduction)	Systemic chemotherapy (e.g., vincristine, etoposide, carboplatin).	Myelosuppression Nausea Hair loss Hearing loss Fever Peripheral neuropathy Acute myelogenous leukemia Risk of infection
Intra-arterial Chemotherapy	Localized delivery via ophthalmic artery.	Eyelid swelling Periocular erythema Retinal artery occlusion Stroke (rare) Mild neutropenia
Intravitreal Chemotherapy	Direct injection into vitreous for vitreous seeding control.	Retinal detachment Endophthalmitis Hemorrhage Retinal atrophy
Periocular Chemotherapy	Injection around the eye to increase local drug levels.	Orbital inflammation Fat atrophy Strabismus
Intracameral Chemotherapy	Injection into the anterior chamber (less common).	Corneal edema Glaucoma Anterior segment inflammation
Tylectomy	Local resection of the tumor (rare, controversial).	Tumor spread (if improper technique), intraocular complications
Chemoplaque	Radioactive plaque + chemotherapy placed on sclera for localized effect.	Localized radiation damage Conjunctival inflammation Scleral necrosis (rare)
